# The prevalence of dengue virus serotypes in asymptomatic blood donors reveals the emergence of serotype 4 in Saudi Arabia

**DOI:** 10.1186/s12985-017-0768-7

**Published:** 2017-06-09

**Authors:** Ahmed Mohamed Ashshi

**Affiliations:** Laboratory Medicine Department, Faculty of Applied Medical Sciences, Umm Al-Qura University, PO Box 7607, Al Abdeyah, Holy Makkah, Kingdom of Saudi Arabia

**Keywords:** Dengue virus, Blood transfusion, Multiplex PCR, Saudi Arabia

## Abstract

**Background:**

Transmission of dengue virus (DENV) through blood transfusion has been documented and hence screening for DENV during blood donation has been recently recommended by the American Association of Blood Banks and Centres of Disease Control and Prevention. DENV is endemic in the Western province of the Kingdom of Saudi Arabia (KSA) and serotypes 1, 2 and 3, but not 4, have been detected. However, little is known regarding the rates of DENV during blood donation in the kingdom. The aim of this study was therefore to measure the prevalence of dengue virus and its serotypes in eligible Saudi blood donors in the endemic Western region of KSA.

**Methods:**

This was a cross-sectional study and serum samples were collected from 910 eligible Saudi male blood donors. DENV IgM and IgG antibodies were measured serologically by ELISA while viral serotypes were detected by a single step IVD CE certified multiplex RT-PCR kit.

**Results:**

The overall prevalence was 39 and 5.5% for IgG+ and IgM+, respectively. There were 12 (1.3%) with exclusively IgM+, 317 (34.8%) exclusively IgG+ and 38 (4.2%) with dual IgM+/IgG+ donors. The overall prevalence was 3.2% (*n* = 29) and 2.3% (*n* = 21) for primary and secondary infections. PCR was positive in 5.5% (*n* = 50) and, DENV-2 (*n* = 24; 48%) was the most frequent serotype and was significantly higher than DENV-1 (20%; *P* = 0.02) and DENV-3 (2%; *P* = 0.1 × 10^−5^) but not DENV-4 (30%; *P* = 0.2). There was no significant difference between both DENV-4 and DENV-1 (*P* = 0.4). The combination of the PCR and serology findings showed that 22 (2.4%) and 28 (3.1%) donors had primary and secondary viremic infections, respectively.

**Conclusions:**

The detected rates of DENV by PCR suggest a potential high risk of viral transmission by blood transfusion. To the best of our knowledge, this study is the first to report the detection of DENV-4 serotype in Saudi Arabia. More studies are required to measure the precise prevalence of DENV serotypes and their potential transmission rate during blood donation in the kingdom.

## Background

Dengue fever is a mosquito-borne viral infection caused by one of the four serotypes of dengue virus (DENV 1–4) and the majority of infected cases are asymptomatic or only report flu-like symptoms even with high viral load [1, 2]. Nonetheless, each DENV serotypes produces a specific immune reactivity and sequential infection with different serotypes is therefore believed to induce more serious pathologies such as dengue haemorrhagic fever and dengue shock syndrome [2].

The first outbreak of dengue infection in the Kingdom of Saudi Arabia (KSA) occurred in Jeddah city in the Western province during 1994 [3, 4]. The virus has later spread to other nearby cities, including Makkah, and the Western region has been declared dengue endemic following several outbreaks throughout the last decade [5, 6]. Additionally, the virus and/or its vector have been found in other new geographical areas of the kingdom as shown by several recent epidemiological reports [7–9]. Phylogenetic studies have also shown that DENV-1, −2 and −3, but not DENV-4, were circulating among symptomatic cases [10–13].

The laboratory diagnosis of dengue is challenging and the results are usually interpreted in light of fever onset in symptomatic patients [1]. The gold standard diagnostic tool of acute dengue infection is viral isolation and culturing in vitro, which is time consuming and not feasible in many clinical laboratories [1, 2]. Therefore, the laboratory diagnosis of acute dengue is currently performed either serologically by capturing the viral non-structural protein-1 (NS1) or by detecting the virus RNA by RT-PCR [2, 14]. However, NS1-based diagnosis, unlike RT-PCR, lacks the advantage of viral serotyping [14].

The American Association of Blood Banks and Centres of Disease Control and Prevention have recently included dengue among the screening panel of pathogens for blood safety since seven clusters of transfusion transmitted dengue have been documented from several endemic countries [15–17]. The virus has also been isolated from the different blood components of acutely infected asymptomatic donors and has been shown to survive and replicate, even during storage, in RBCs and platelets [18–20]. However, screening for dengue during blood donation is yet not recommended by the health authorities in KSA even in the endemic regions [21].

Our research team has previously reported in a pilot study a rate of 5% for NS1 in asymptomatic blood donors and who were eligible for blood donation [21]. This study was therefore conducted to measure the rates and serotypes of DENV RNA by an IVD TaqMan multiplex assay and the results were correlated with those of DENV IgM and IgG antibodies. A better understanding about the prevalence of DENV and its serotypes among asymptomatic blood donors in the endemic regions could help the policy makers in the kingdom to develop a safer environment for blood transfusion.

## Methods

### Ethical approval

Ethical approval was obtained from the Faculty of Applied Medical Sciences Ethics Committee concomitantly with an official governmental approval that was secured in writing by the Deputy Director of Health of the Holy Makkah Municipality. All serum samples were collected following obtaining informed written consent from all the participants.

### Study design

This was a cross-sectional study and a total of 910 apparently healthy and eligible Saudi male blood donors with age ranging between 25 and 55 years (mean 37.13 ± 7.45 years) were recruited from the blood banks of Hira General Hospital and The Regional Laboratory, Holy Makkah, in the Western province of KSA between March 2015 and August 2016. All participants had no history of fever in the preceding 3 weeks of donation or any other sign of dengue infection according to the WHO guidelines [21]. The donors were also serologically negative for human immunodeficiency, hepatitis B, and hepatitis C viruses; and were eligible for blood donation as per to the policy of the KSA Ministry of Health. Serum was obtained from each donor following centrifugation of 10 mL of venous blood that was collected in sterile plain tubes without anticoagulant. All samples were aliquoted in small volumes of 500 μl each and stored in −80 °C and −20 °C until used for PCR and ELISA, respectively.

### Enzyme linked immunosorbant assay (ELISA)

ELISA was used for the qualitative detection of IgM and IgG antibodies against DENV and the used kits (Panbio, Brisbane, QLD, Australia) are certified for clinical in vitro diagnosis (IVD CE) and were from the same batch. All samples and the internal controls provided within each kit were processed according to the manufacturer’s instructions on a fully automated ELISA system (Human Diagnostics, Germany). The sensitivity, specificity, inter and intra-assay coefficient of variation for each kit as reported by the manufacturer were 97.7, 100, 8.8 and 9.8% for the IgG kit and, 94.7, 100, 7.8 and 5.6% for the IgM kit. The cut-off value for each kit was calculated followed by the calculation of the index values for each sample as per the manufacturer’s instructions. Samples were considered positive if their values for IgM and/or IgG were > (cut-off + 10% cut-off value). The participants were classified into primary infection or previous exposure groups according to the solitary detection of IgM or IgG antibodies, respectively. In the case of dual positivity for both antibodies, the donors were categorised as either primary or secondary infection groups based on the IgM/IgG ratio, where a ratio ≥ 1.2 suggested primary while < 1.2 indicated a secondary infection [22].

### Real-time TaqMan PCR for the detection of DENV serotypes

One step Multiplex TaqMan RT-PCR for the qualitative detection and differentiation of DENV-1, 2, 3 and 4 was performed by using the IVD CE certified FTD Dengue differentiation kit (Fast-track diagnostics, Junglinster, Luxembourg) on ABI® 7500 platform (Thermo Fisher Scientific) and according to the manufacturer’s protocol. As reported by the manufacturer, the kit has a detection limit of 10^3^ copies/ml for all serotypes except DENV-3, which could be detected until 10^2^ copies/ml. However, the kit does not measure the viral load of the candidate viral serotypes.

The internal control provided by the manufacturer was brome mosaic virus, which was added at the lysis buffer stage with each serum sample during the RNA extraction process. The extraction of viral RNA from the collected serum samples was carried-out using MagMAX™-96 AI/ND Viral RNA Isolation Kit (Thermo Fisher Scientific, Warrington, UK) according to the manufacturer’s instructions on a MagMAX™ Express Magnetic Particle Processor (Thermo Fisher Scientific). The quality and quantity of extracted RNA were measured on the BioSpec-nano (Shimadzu Corporation, Tokyo, Japan) and typically had an A260/A280 ratio of 1.7 to 1.9. Extracted RNA samples were stored at −80 °C until used.

Proper isolation of nucleic acids was assured by the successful amplification of the internal control, which also confirmed the absence of PCR inhibitors. An extra in-house validation protocol was also performed for all negative samples by spiking them with the provided positive RNA controls at 4:1 ratio by adding 2 μl of positive controls to 8 μl sample. A detection of a signal following this step was reassuring that the designated viral serotypes were not present in the original negative samples as previously described [23]. The PCR reaction per well consisted of 12.5 μl mastermix, 1 μl enzyme, 1.5 μl of primers and 10 μl RNA. The amplification was performed as instructed by the manufacturer. The validation of the results was performed according to the manufacturer’s guidelines and by using the provided positive and negative controls within the kit.

The study participants were re-classified following the integration of serology and PCR results into negative, viremic 1^ry^ infection, convalescent-phase 1^ry^ infection, viremic 2^ry^ infection, convalescent-phase 2^ry^ infection and previous exposure groups according to the criteria listed in Table 1.

**Table 1:** Diagnostic classification of the study participants according to their DENV serological and PCR results

Group	Laboratory diagnostic criteria
Negative group	Negative by all serological and PCR tests
Viremic 1^ry^ infection group	Positive PCR ± IgM positive antibodies
Convalescent-phase 1^ry^ infection group	Either exclusively positive for IgM or dual positive IgM and IgG antibodies with an IgM/IgG ratio ≥ 1.2
Viremic 2^ry^ infection group	Simultaneously positive PCR with positive IgG ± IgM antibodies
Convalescent-phase 2^ry^ infection	Negative PCR and dual positive IgM and IgG antibodies with an IgM/IgG ratio < 1.2
Previous exposure group	Exclusively positive for IgG antibodies

### Statistical analysis

Statistical analysis of the results was performed with SPSS version 16. Categorical variables were compared by Chi square (*χ* ^2^) or Fisher’s tests following cross-tabulation and significance was considered if P was < 0.05.

## Results

### Frequency of dengue infection by serology

**Fig. 1:**
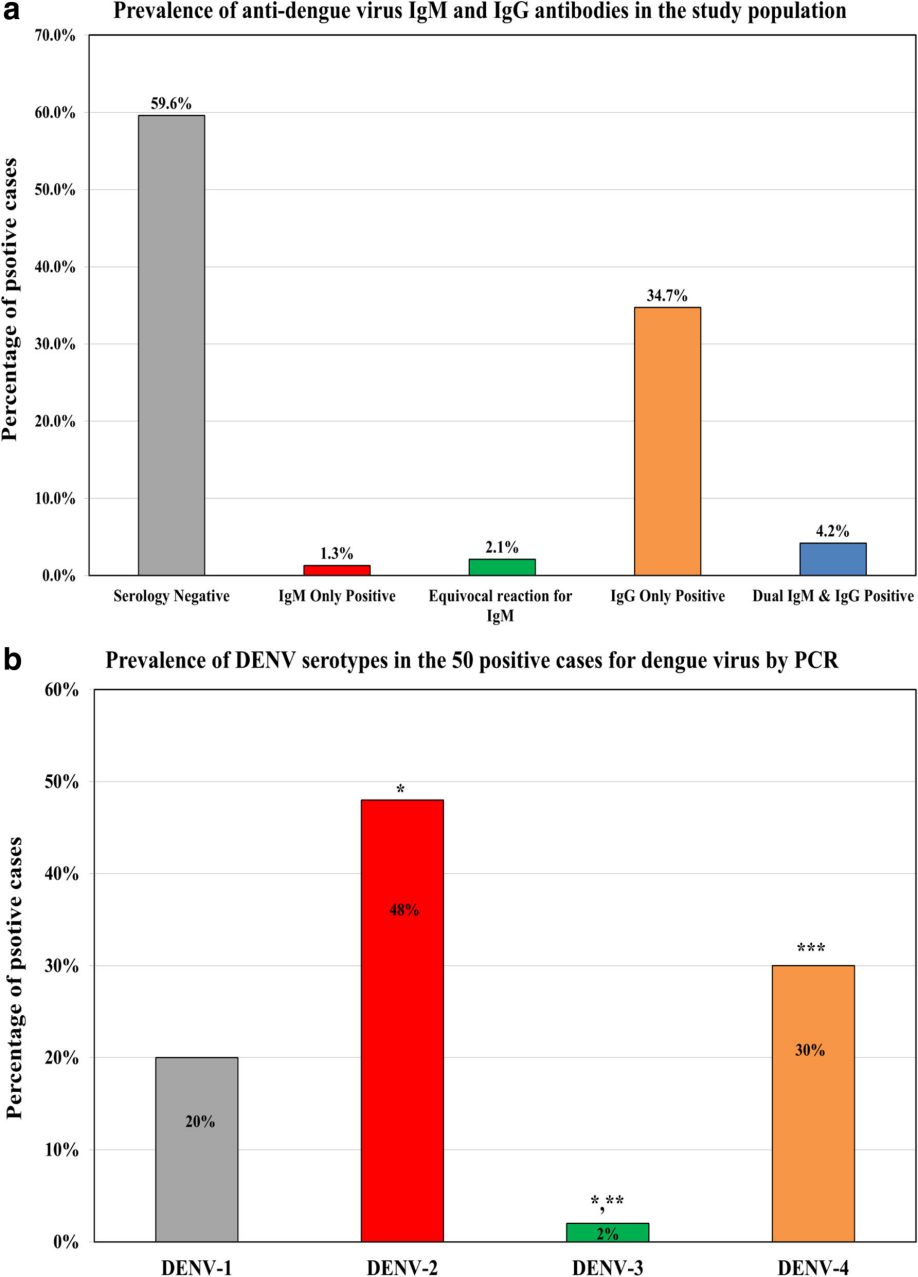
The prevalence of (**a**) anti-dengue IgM and IgG antibodies among the 910 study participants and (**b**) DENV serotypes in the 50 cases positives by PCR. (* = *P* < 0.05 compared with DENV-1; ** = *P* < 0.05 compared with DENV-2 and *** = *P* < 0.05 compared with DENV-3)

The overall rates of detecting anti-dengue antibodies was 5.5% (*n* = 50) by IgM and 39% (*n* = 355) by IgG. There were 38 donors with dual positivity for both IgM and IgG antibodies together with 12 sole IgM and another 317 exclusively IgG positive cases (Fig. 1a). According to the IgM/IgG ratio of those participants with dual positive antibodies (*n* = 38), 17 (44.7%) had primary infection and the remaining (55.3%) were classified as secondary infection. Equivocal reaction for IgM was detected in 19 subjects (2.1%) and none of the participants showed equivocal reaction for the IgG antibodies. The overall prevalence was 3.2% (*n* = 29) for primary infection and 2.3% (*n* = 21) for secondary infection by serology.

### Prevalence of DENV serotypes

The multiplex-PCR assay clearly distinguished and identified all four DENV serotypes in the serum samples and no co-infection was observed between the different serotypes. Signals were also detected in all wells that included the provided positive controls with no signal with the negative controls. Spiked negative serum samples with the provided RNA of DENV serotypes also showed a positive signal reassuring the observed results of this study.

Dengue virus was positive by PCR in 5.5% (*n* = 50) of the 910 study participants and the most frequent serotype was DENV-2 (*n* = 24) and it was significantly higher than DENV-1 (*n* = 10; *P* = 0.02) and DENV-3 (*n* = 1; *P* = 0.1 × 10^−5^) but not DENV-4 (*n* = 15). Additionally, there was no significant difference between both DENV-4 and DENV-1 serotypes. The frequencies of DENV serotypes in relation to the serological results are summarised in Fig. 1b.

**Fig. 2:**
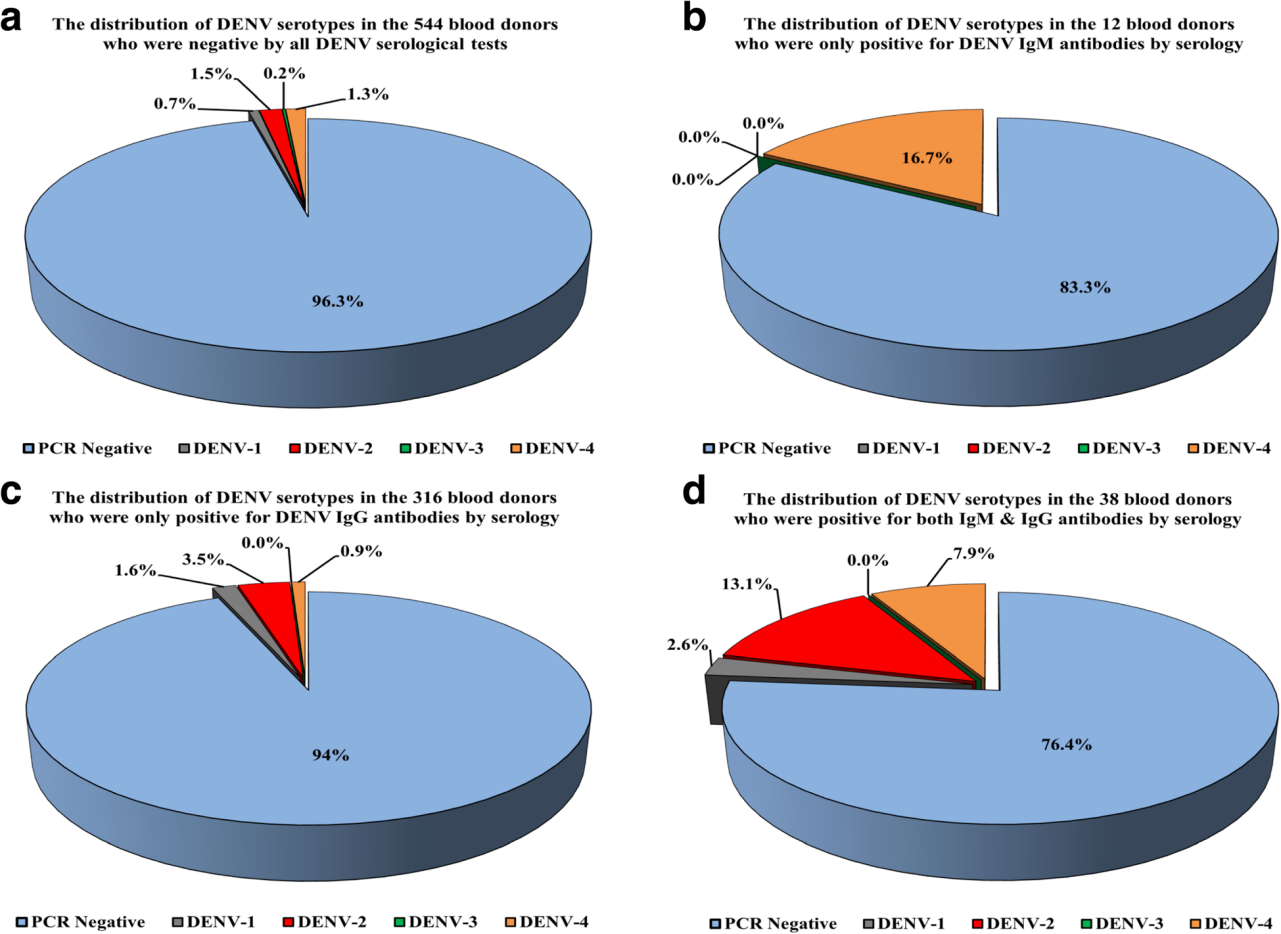
The distribution of DENV serotypes in the study participants who were (**a**) serologically negative by all test, (**b**) exclusively positive for IgM antibodies, (**c**) exclusively positive for IgG antibodies and (**d**) dual positive for IgM and IgG antibodies

By analysing the frequencies of DENV serotypes in view of the serological results, DENV-2 was significantly more prevalent than DENV-1 (*P* = 0.04) and DENV-3 (*P* = 0.02) serotypes, but not DENV-4, in those participants (*n* = 544) who were serologically negative for both IgM and IgG antibodies (Fig. 2). Similarly, the frequencies of DENV-2 were significantly higher than all other serotypes in those donors who were either only IgG+ or showed dual positivity for both antibodies, but was not detected in those donors with exclusive IgM+ (Fig. 2). DENV-4 was not significantly different from DENV-1 and DENV-3 in all groups except in the serology negative where it was significantly higher (*P* = 0.03) than serotype 3 (Fig. 2).

The integration of the PCR findings with the serological observations showed that 524 donors (57.6%) were negative by all diagnostic tests, 22 (2.4%) had primary viremic infection, 28 (3.1%) with secondary viremic infection, 27 (3%) with convalescent primary infection, 12 (1.3%) convalescent secondary infection and 297 (32.6%) with a previous exposure. The frequencies of DENV serotypes in the primary (*n* = 22) and secondary (*n* = 28) viremic infection groups are summarised in Table 2.

**Table 2:** Distribution of DENV serotypes among those blood donors diagnosed with primary and secondary viremic infections

	DENV Serotypes by PCR				Total (%)
	*DENV-1 (%)*	*DENV-2 (%)*	*DENV-3 (%)*	*DENV-4 (%)*	
*Primary viremic infection*	4 (8)	8 (16)	1 (2)^b^	9 (18)^d^	22 (44)
*Secondary viremic infection*	6 (12)	16 (32)^a^	ND (0)^a,c^	6 (12)^b,d^	28 (56)
Total (%)	10 (20)	24 (48)^a^	1 (2)^a,b^	15 (30)^d^	50 (100)

^ND^ = not detected

^a^ = *P* < 0.05 compared with DENV-1

^b^ = *P* < 0.05 compared with DENV-2

^c^ = *P* < 0.01 compared with DENV-2

^d^ = *P* < 0.05 compared with DENV-3

## Discussion

Herein, the prevalence of dengue virus and its serotypes was measured by a single step multiplex RT-PCR together with IgM and IgG antibodies in serum samples for the diagnosis of asymptomatic acute dengue infection in 910 eligible Saudi blood donors. The results showed a high rate of previous exposure (32.6%) among the study population in addition to 5.5% frequency of acute dengue infection among blood donors in KSA as shown by the results of IgG and IgM antibodies, respectively. These observations are in agreement with our previous pilot data that has demonstrated a similar seroprevalence among blood donors from the same geographical area [21].

To the best of our knowledge, this is the first report on the different DENV serotypes in asymptomatic blood donners in KSA and the results showed an overall 5.5% prevalence of dengue by PCR, similar to the results of anti-dengue IgM antibodies, and the most prevalent serotype was DENV-2 and the lowest detected was DENV-3 serotype. These observations are in agreement with the previously published studies from the kingdom, where similar rates of dengue viral serotypes −1, −2 and −3 have been continuously observed in the Western province of the country since the first epidemic in 1994 [3, 12, 24]. Furthermore, the current data suggest that many of the IgM positive cases (*n* = 50) were at the convalescence stage of infection since not all individuals were concomitantly positive by PCR [1, 14].

Interestingly, this study is also the first to report DENV-4 in KSA and the rate of detecting this serotype was similar to those of DENV-2, which is the most prevalent serotype in Jeddah and Makkah cities [4, 6, 10, 12, 25, 26]. Moreover, DENV-4 was the only detected serotype among those individuals who were exclusively positive for IgM antibodies. Collectively, these results suggest the recent introduction of DENV-4 serotype since the most recent phylogenic study from the same region, which retrospectively examined all dengue positive samples between 2010 and 2015, has only reported DENV 1–3 serotypes [11].

A possible explanation for the present findings could be the high numbers as well as the diversity of travelers visiting the area each year for pilgrimage and/or the presence of a large population of immigrant workers from dengue-endemic countries [12, 27]. In this context, several studies have shown independent multiple introductions of DENV-1 (lineage India-2 and lineage Asia-2) and DENV-2 (Cosmopolitan genotype and DENV-2-Jeddah-2014) in the Western province of the kingdom between the years 1994 and 2014 [10, 11, 25]. Additionally, the most recent report by the United Nations has estimated that 10 million migrant workers are living in KSA and many of them are from India and Pakistan where DENV-4 is endemic [28–30]. Additionally, there was no significant difference in the present study between the frequencies of DENV-4 and −1, and the latter has also recently been isolated for the first time from symptomatic patients in Makkah city [26]. Coherently, other research groups have also demonstrated the spread of dengue and its vector into other regions of the kingdom and in which they were previously absent [7–9]. Therefore, the present data support the many previous calls for the health authorities in KSA to implement a continuous vigilance program to monitor the circulation of dengue virus together with the urgent needs for more effective vector control measures, especially during the pilgrimage season, in order to prevent the introduction of new DENV strains and/or elimination of viral spread [12, 27].

In parallel with the previous findings, the present study also demonstrated a high rate of a previous exposure among the study participants, which is similar to the previously reported rates of this viral infection in the kingdom and reflects on the endemic nature of dengue in the region [3, 5, 8, 10, 12, 13, 26, 27]. Additionally, the frequency of viremia, as indicated by the findings of PCR, was 5.5% in asymptomatic blood donors and the majority of cases were classified as secondary infection (56%). This study therefore provides further support to the notion that blood transfusion could facilitate the transmission of dengue virus, particularly in endemic countries [15–19, 21].

In this regards, seven clusters of dengue transmission through blood transfusion have been previously documented in endemic countries and/or during an outbreak of dengue infection [15–17, 31, 32]. Additionally, it has been shown that dengue virus can replicate in RBCs and platelets together with surviving within these cells during storage in blood banks [18–20]. A transfusion rate of 37.5% has also been estimated by a very recent study among blood donors in Brazil following an outbreak of DENV-4 during 2012 [32]. Nevertheless, screening for dengue acute/viremic infection represent a diagnostic challenge since it is mainly dependent on the correlation of NS1 and PCR results with the onset of fever, which is frequently not reported by the majority of acutely infected blood donors [8, 9, 13, 23, 25]. Furthermore, none of the two markers of viremic stage could achieve individually a 100% sensitivity due to their short life span in plasma [1, 2, 14, 22].

The present study is in agreement with the previous reports and provides further evidence regarding the potential threat of dengue transmission by blood transfusion in endemic regions. In recent years the source of blood in Saudi blood banks has dramatically shifted from absolute importation towards local recruitment of donors from Saudi and migrant populations in the kingdom, who cover almost 90% of the national blood banks requirements [33, 34]. However, there is no official report on the annual frequency and amount of blood donation in KSA but a recent announcement in a local newspaper reported the collection of 354,633 units by the Ministry of Health in 2016, among which 63,610 units (18%) were collected from Makkah city [35]. Therefore, more studies are needed to be conducted in the Western province of KSA to measure the factual rates of asymptomatically infected blood donors at the viremic stage in the different cities of endemic regions within the kingdom in order to develop appropriate measures for the prevention of dengue transmission through blood transfusion.

There are several limitations to the present study. The PCR results were not confirmed/complemented by another diagnostic technique (e.g., NS1 and virus isolation), which could have led to an underestimation in the rates of asymptomatic viremic donors. Additionally, all donors in the present study were males since females in KSA do not donate blood mainly due to social and cultural barriers [34]. Furthermore, we were not able to reflect on the viral load among those PCR positive individuals since the used kit does not measure the viral load, which could had been a reason for the asymptomatic nature of the detected infections. Another limitation is related to the non-tracing of recipients to measure the transmission rate through blood transfusion. Finally, the month/season of sample collection was not given by the blood banks for the majority of cases and therefore the research team was not able to identify the peak season for potential transmission. Hence, more studies are mandatory and they should measure the frequencies of acute dengue infection among blood donors together with recipients by more than one method and to record the time of donation to accurately reflect the actual magnitude of possible dengue transmission through blood donation in this endemic regions of KSA.

## Conclusions

The observed high rates of viremic but asymptomatic blood donors in the endemic Western region of KSA suggest that the recipients are potentially at risk of acquiring dengue infection by blood transfusion, which could be prevented by the inclusion of a screening program for this viral infection during blood donation. Additionally, this is the first report on the emergence of DENV-4 serotype in the kingdom, postulating that the currently applied measures for the prevention of dengue infection are not sufficient and require further stringent methods in order to successfully control/prevent the spread of this viral infection. Further studies are needed to measure the factual prevalence of dengue virus and its transmission rates during blood donation in the different regions of the kingdom.

## Abbreviations

DENV: Dengue virus
KSA: Kingdom of Saudi Arabia
NS1: Non-structural protein-1
RT-PCR: Reverse transcription polymerase chain reaction
WHO: World Health Organization

## References

[1] Bhat VG, Chavan P, Ojha S, Nair PK (2015). Challenges in the Laboratory Diagnosis and Management of Dengue Infections. Open Microbiol J.

[2] Ho TS, Wang SM, Lin YS, Liu CC (2013). Clinical and laboratory predictive markers for acute dengue infection. J Biomed Sci.

[3] Fakeeh M, Zaki AM (2001). Virologic and serologic surveillance for dengue fever in Jeddah, Saudi Arabia, 1994–1999. Am J Trop Med Hyg.

[4] Fakeeh M, Zaki AM (2003). Dengue in Jeddah, Saudi Arabia, 1994–2002. Dengue Bull.

[5] Khan NA, Azhar EI, El-Fiky S, Madani HH, Abuljadial MA, Ashshi AM, Turkistani AM, Hamouh EA (2008). Clinical profile and outcome of hospitalized patients during first outbreak of dengue in Makkah, Saudi Arabia. Acta Trop.

[6] Alhaeli A, Bahkali S, Ali A, Househ MS, El-Metwally AA (2016). The epidemiology of Dengue fever in Saudi Arabia: A systematic review. J Infect Public Health.

[7] Al-Azraqi TA, El Mekki AA, Mahfouz AA (2013). Seroprevalence of dengue virus infection in Aseer and Jizan regions, Southwestern Saudi Arabia. Trans R Soc Trop Med Hyg.

[8] El-Badry A, Al-Ali K (2010). Prevalence and seasonal distribution of dengue mosquito, Aedes aegypti (Diptera: Culicidae) in Al-Madinah Al-Munawwarah, Saudi Arabia. J Entomol.

[9] Kheir SM, Alahmed AM, Al Kuriji MA, Al Zubyani SF (2010). Distribution and seasonal activity of mosquitoes in al Madinah Al Munwwrah, Saudi Arabia. J Egypt Soc Parasitol.

[10] Zaki A, Perera D, Jahan SS, Cardosa MJ (2008). Phylogeny of dengue viruses circulating in Jeddah, Saudi Arabia: 1994 to 2006. Trop Med Int Health.

[11] Al-Saeed MS, El-Kafrawy SA, Farraj SA, Al-Subhi TL, Othman NA, Alsultan A, Ben Helaby HG, Alshawdari MM, Hassan AM, Charrel RN, Azhar EI, Hashem AM (2017). Phylogenetic characterization of circulating Dengue and Alkhumra Hemorrhagic Fever viruses in western Saudi Arabia and lack of evidence of Zika virus in the region: A retrospective study, 2010–2015. J Med Virol.

[12] Aziz AT, Al-Shami SA, Mahyoub JA, Hatabbi M, Ahmad AH, Rawi CS (2014). An update on the incidence of dengue gaining strength in Saudi Arabia and current control approaches for its vector mosquito. Parasit Vectors.

[13] Jamjoom GA, Azhar EI, Kao MA, Radadi RM (2016). Seroepidemiology of Asymptomatic Dengue Virus Infection in Jeddah, Saudi Arabia. Virology (Auckl).

[14] Ahmed NH, Broor S (2014). Comparison of NS1 antigen detection ELISA, real time RT-PCR and virus isolation for rapid diagnosis of dengue infection in acute phase. J Vector Borne Dis.

[15] Levi JE (2016). Dengue Virus and Blood Transfusion. J Infect Dis.

[16] Walsh GM, Shih AW, Solh Z, Golder M, Schubert P, Fearon M, Sheffield WP (2016). Blood-Borne Pathogens: A Canadian Blood Services Centre for Innovation Symposium. Transfus Med Rev.

[17] Arellanos-Soto D, BdlC V, Mendoza-Tavera N, Ramos-Jimenez J, Cazares-Tamez R, Ortega-Soto A, Rivas-Estilla AM (2015). Constant risk of dengue virus infection by blood transfusion in an endemic area in Mexico. Transfus Med.

[18] Simon AY, Sutherland MR, Pryzdial EL (2015). Dengue virus binding and replication by platelets. Blood.

[19] Sutherland MR, Simon AY, Serrano K, Schubert P, Acker JP, Pryzdial EL (2016). Dengue virus persists and replicates during storage of platelet and red blood cell units. Transfusion.

[20] Anez G, Heisey DA, Chancey C, Fares RC, Espina LM, Souza KP, Teixeira-Carvalho A, Krysztof DE, Foster GA, Stramer SL, Rios M (2016). Distribution of Dengue Virus Types 1 and 4 in Blood Components from Infected Blood Donors from Puerto Rico. PLoS Negl Trop Dis.

[21] Ashshi AM (2015). Serodetection of Dengue virus and its antibodies among blood donors in the western region of Saudi Arabia: a preliminary study. Blood Transfus.

[22] De La Cruz Hernández SI, Reyes-del Valle J, Villegas-del Angel E, Ludert JE, del Angel RM (2015). Dengue laboratory diagnosis: still some room for improvement. Futur Virol.

[23] Ashshi AM, Batwa SA, Kutbi SY, Malibary FA, Batwa M, Refaat B (2015). Prevalence of 7 sexually transmitted organisms by multiplex real-time PCR in Fallopian tube specimens collected from Saudi women with and without ectopic pregnancy. BMC Infect Dis.

[24] Alzahrani AG, Al Mazroa MA, Alrabeah AM, Ibrahim AM, Mokdad AH, Memish ZA (2013). Geographical distribution and spatio-temporal patterns of dengue cases in Jeddah Governorate from 2006–2008. Trans R Soc Trop Med Hyg.

[25] El-Kafrawy SA, Sohrab SS, Ela SA, Abd-Alla AM, Alhabbab R, Farraj SA, Othman NA, Hassan AM, Bergoin M, Klitting R, Charrel RN, Hashem AM, Madani TA, Azhar EI (2016). Multiple Introductions of Dengue 2 Virus Strains into Saudi Arabia from 1992 to 2014. Vector Borne Zoonotic Dis.

[26] Organji SR, Abulreesh HH, Osman GEH (2017). Circulation of Dengue Virus Serotypes in the City of Makkah, Saudi Arabia, as Determined by Reverse Transcription Polymerase Chain Reaction. Can J Infect Dis Med Microbiol.

[27] Amarasinghe A, Letson GW (2012). Dengue in the Middle East: a neglected, emerging disease of importance. Trans R Soc Trop Med Hyg.

[28] United Nations DoEaSA. Trends in international migration, 2015. Population Facts 2015; Available from: http://www.un.org/en/development/desa/population/publications/pdf/popfacts/PopFacts_2015-4.pdf. Accessed 20 May 2017.

[29] Saha K, Ghosh M, Firdaus R, Biswas A, Seth B, Bhattacharya D, Mukherjee K, Sadhukhan PC (2016). Changing pattern of dengue virus serotypes circulating during 2008–2012 and reappearance of dengue serotype 3 may cause outbreak in Kolkata, India. J Med Virol.

[30] Suleman M, Lee HW, Zaidi SS, Alam MM, Nisar N, Aamir UB, Sharif S, Shaukat S, Khurshid A, Angez M, Umair M, Mujtaba G, Faryal R (2017). Preliminary Seroepidemiological survey of dengue infections in Pakistan, 2009–2014. Infect Dis Poverty.

[31] Stramer SL, Linnen JM, Carrick JM, Foster GA, Krysztof DE, Zou S, Dodd RY, Tirado-Marrero LM, Hunsperger E, Santiago GA, Munoz-Jordan JL, Tomashek KM (2012). Dengue viremia in blood donors identified by RNA and detection of dengue transfusion transmission during the 2007 dengue outbreak in Puerto Rico. Transfusion.

[32] Busch MP, Sabino EC, Brambilla D, Lopes ME, Capuani L, Chowdhury D, McClure C, Linnen JM, Prince H, Simmons G, Lee TH, Kleinman S, Custer B, International Component of the NRE, Donor Evaluation S, III (2016). Duration of Dengue Viremia in Blood Donors and Relationships Between Donor Viremia, Infection Incidence and Clinical Case Reports During a Large Epidemic. J Infect Dis.

[33] Abdel Gader AG, Osman AM, Al Gahtani FH, Farghali MN, Ramadan AH, Al-Momen AK (2011). Attitude to blood donation in Saudi Arabia. Asian J Transfus Sci.

[34] Al-Johar AW, Al-Saud A, Abalkhail Y, Jawdat T, Al-Khamees S, Al-Thunayan F, Abdel-Gader AG (2016). Why do-Saudi Women Refrain Donating Their Blood?-a Study on the Attitude, Belief and Motivation of Saudi Female University Students Towards Blood Donation. Clin Lab.

[35] MoH blood bank collects 354,633 units in one year. Arab News 2016; Available from: http://www.arabnews.com/node/963921/saudi-arabia. Accessed 20 May 2017.

